# Competencies and needs of nurse educators and clinical mentors for teaching in the digital age – a multi-institutional, cross-sectional study

**DOI:** 10.1186/s12912-022-01018-6

**Published:** 2022-08-28

**Authors:** Stefan Jobst, Ulrike Lindwedel, Helga Marx, Ronja Pazouki, Sven Ziegler, Peter König, Christiane Kugler, Johanna Feuchtinger

**Affiliations:** 1 Institute of Nursing Science, Faculty of Medicine, University of Freiburg, Freiburg, Germany; 2 Center of Implementing Nursing Care Innovations, Freiburg, Germany; 3 Care and Technology Lab, Furtwangen University of Applied Sciences, Furtwangen, Germany; 4 University Medical Center, Freiburg, Germany

**Keywords:** Nursing education, Nurse educator, Clinical mentor, Digital technology, Digital competence, Survey, Continuing education

## Abstract

**Background:**

The impact of technology and digitalization on health care systems will transform the nursing profession worldwide. Nurses need digital competencies to integrate new technology in their professional activities. Nurse educators play a crucial role in promoting the acquisition of digital competences and therefore need to be digitally competent themselves. Research on digital competencies of nursing educators is scarce but suggests lack of digital knowledge and skills and support needs. Although digitalization is to be seen as a global process, regional contexts need to be taken into account, such as pre-existing competencies, local conditions, and individual needs. Thus, it remains unclear which competencies nurse educators possess and which support needs they have.

Aim of this study was to assess nurse educators’ and clinical mentors’ digital competencies and explore their needs and requirements concerning the digital aspects of their pedagogy and teaching activities in Germany.

**Methods:**

A descriptive exploratory study with a cross-sectional design was conducted. Participants were identified using a convenience sampling approach. Data were collected during July and September 2020 using a standardized self-reported questionnaire that was developed specifically for this study. The questionnaire was provided in a paper and online format and participants could decide which format to use. It contained open- and closed-ended questions. Data were analyzed using descriptive and content analysis. Additionally, explorative subgroup analyses based on job designation, age, and gender were performed. Reporting of this study adhered to the STROBE checklist.

**Results:**

A total of 169 educating nurses participated in the survey. The respondents considered themselves as digitally competent and showed a positive attitude towards the integration of digital technology in their teaching activities. Their perceived preparedness to integrate digital technology into teaching and training varied. Almost all respondents (98%) declared a need for further training and seemed motivated to participate in corresponding educational events. There were some indications for differences in competencies or needs between subgroups.

**Conclusions:**

Educating nurses appear to possess basic digital competencies but there is a need to support their professional development in terms of new technologies. Findings can be used as a basis for developing supportive interventions. Further qualitative investigations could inform the design and content of such interventions.

## Background

Technology and digitalization have a major impact on health care systems and will transform the nursing profession worldwide [1]. A cornucopia of digital technologies already exists for nursing care – some widely implemented in practice (e.g. electronic health records), others still new and unexplored (e.g. robotics) [2]. In Germany, the digitalization of healthcare has even been established by law since 2020 with the “Digital Healthcare Act” and the “Act for the Digital Modernisation of Care and Nursing”. Therefore, nurses are in need for digital competencies to integrate and apply new technology in their professional activities [3].

There is no uniform definition of competence [4]. It can be seen as the composition of individual competencies in the areas of knowledge, skills, and, most often, attitudes or values [5]. *Digital* competence is an umbrella term that “describes a network of intricately connected purposes, domains, and levels of ICT [information and communication technology] use” [6] and can be defined as “the set of knowledge, skills, attitudes, abilities, strategies and awareness that is required when using ICT and digital media” [7]. In the context of this study competence was broadly operationalized as being composed of knowledge, skills and attitude.

For nurses, the foundations for these digital competencies are acquired during their vocational training/undergraduate education, ongoing training, and are deepened in their workplaces. In this process, nurse educators play a crucial role in facilitating learning with technology [8] to support a “successful technological evolution” [9]. Therefore, educators themselves should be able to integrate the necessary digital competencies into their pedagogical work [9, 10]. This can be referred to as pedagogical digital competence that “involves all kinds of pedagogical work in professional contexts where digital technology is used” [11].

Digital technologies in healthcare education can be classified into mobile technologies, e-learning, simulation, and classroom-response systems [12]. Various technologies focus on both theoretical teaching in the classroom and practical training. Studies showed that mobile technologies, for example, enabled access to training content according to individual needs and supported and improved interaction with educators [13]. Voutilainen et al. [14] discovered that e-learning in certain situations, had a positive effect on the learning outcomes of nursing students in comparison to conventional teaching.

Consequently, the competencies that nurse educators must impart to their students for daily practice are increasing significantly [15]. It is essential that nurse educators remain abreast of these trends by including technologies into class work while also receiving appropriate support [16].

Evidence addressing the digital competence of healthcare educators is scarce [11, 17]. Kinnunen et al. [16] outlined competence areas recommended for nurse educators. Forman et al. [11] concluded that there was no consensus about a minimal digital competence level, and that there were barriers to the integration of technology and a requirement for more support using technology in education. In a recent Finnish survey, Männistö et al. [10] argued that competence was a complex issue and seemed to be related to different factors. For German speaking countries, Egbert et al. [18] compiled nursing informatics core competencies for different fields within the nursing profession (i.e. nursing management, IT management in nursing, quality management, clinical nursing, coordination of inter-professional care), but did not explicitly list the role of nurse educators, stating that these should have the same competencies as those roles in focus of their teaching.

Several studies reported a lack of knowledge and skills related to educational technologies in healthcare educators [19, 20] which directly affects education for nursing students. Shin et al. (2018) [21] recommended to integrate competencies in digital technologies into nursing curricula. However, this means that educating nurses’ digital competence needs to be developed, monitored and updated [10, 16, 22]. Although digitalization is to be seen as a global process, competencies and type of support needs of educating nurses may differ regionally and depend on a variety of factors, such as pre-existing competencies, local conditions, and individual needs [23]. Apart from the aforementioned studies from Scandinavia and North America, studies on digital literacy and the associated support needs of nursing educators in relation to the German context could not be found.

In Germany, pre-registration nursing education is a 3-year hospital-based apprenticeship with theoretical blocks being taught in affiliated schools of nursing. Practical training occurs during placements on various hospital wards and also in other clinical settings [24]. Nurse educators are nurses with at least a bachelor’s degree in nursing education, provide instruction and teaching in nursing schools. Clinical mentors provide supervision during the clinical placements for individual or small groups of students. These nurses have at least one year of professional experience and an additional pedagogical qualification. They are usually employed in the inpatient setting, and integrate their educational role within their clinical responsibilities. Despite the somewhat different orientation of the two professional profiles, the terms ‘nurse educator’ and ‘clinical mentor’ were subsumed under the umbrella term ‘educating nurses’ for better readability throughout this article. Nevertheless, when necessary, the two job designations are considered separately to illustrate differences.

## Methods

### Aim

The purpose of this study was to collect data on the competencies of educating nurses concerning their teaching and training activities in the digital age. The aims were: (1) to assess digital competencies and (2) to explore needs and requirements concerning the digital aspects of pedagogy and teaching activities of educating nurses in four local institutions. Reporting of this study was guided by the Strengthening the Reporting of Observational Studies in Epidemiology (STROBE) Statement [25].

### Design and setting

A descriptive exploratory study with a cross-sectional survey design was conducted at the Freiburg University Medical Centre, the University Cardiac Centre Freiburg, Bad Krozingen, and the two respective affiliated nursing schools.

### Participants

The target group of the survey consisted of all educating nurses (clinical mentors and nurse educators, *N* = 325) at aforementioned centers and schools. Potential participants were identified using a convenience sampling approach via inquiries to gatekeepers (e.g., ward and school managers; subject leaders) at the respective institutions. These provided names and official/institutional mailbox and e-mail addresses, which were used to contact the target group in writing. Sample size calculation was not performed since the total number of educating nurses in Germany necessary for sample size calculation could not be determined. Instead, this study focused on the collection of descriptive exploratory data in one local setting. Incentives for study participation were not provided but respective institutions allowed the questionnaire to be completed during working hours.

### Data collection

A comprehensive review of the literature could not identify a suitable assessment instrument collecting data on digital competencies of educating nurses in German language. Therefore, the authors developed a questionnaire specifically for the study purposes based on (1) national and international theoretical literature on (digital) competencies of nurse educators [26, 27] or educators in general [28, 29], and (2) the analysis of existing assessment instruments on the topic of digital competencies in (nursing) education [30–35]. Four items on general digital competence were based on the TA-EG questionnaire to assess technology affinity [36] in the same wording but with different scaling.

Pretesting of the newly developed instrument was carried out by means of a self-created written questionnaire appraisal tool with closed-ended (scales) and open-ended (free text) questions. Fifteen nursing professionals with teaching experience or current professional activity in education (practical or theoretical training) who did not belong to the target group of the study, confirmed the comprehensibility, readability, clarity and attractiveness of the layout of the questionnaire, and commented on general aspects. The mean time for completion of the questionnaire was 11 min. Only minor revisions (wording, layout) were necessary. The revised version was used in this study.

This standardized self-reported questionnaire consisted of 80 items (open-and closed-ended questions) on five domains covering various aspects (Table 1). Thirty-three of these items were optional, i.e. either subscales with filter questions or scales to be self-defined.

**Table 1:** Overview of structure and content of the questionnaire to assess digital competencies and needs of educating nurses

**Domaine** *• ****Aspect(s) in focus***	**Number of items**	**Response scale (number of items using that scale)**	**Example**
Digital competence in general *• Competence: Knowledge* *• Competence: Skill*	14 *optional:* 6	*•* 4-point Likert-type (4) *•* 6-point semantic distance scale (10) *optional:* *•* written responses (qualitative data) (3) with subscale (6-point semantic distance scale)	“I think I know most of the functions of the electronic devices that I own. (*does not apply at all—rather does not apply—rather applies—fully applies*)”
Digital competence related to teaching and training activities *• Competence: Attitude* *• Competence: Knowledge* *• Competence: Equipment and use*	24 *optional:* 24	*•* single-choice (20) *•* 4-point Likert-type (3) *•* 5-point numeric rating scale (1) *optional:* *•* written responses (qualitative data) (1) *•* written responses (qualitative data) (2) with subscale (4-point frequency scale) *•* 4-point frequency scale (18)	“Technology as a didactic tool: How important do you think it is to integrate digital technologies into teaching? (*unimportant—less important—rather important—very important*)”
Requirements and needs *• Requirements* *• Needs* *• Motivation*	3 *optional:* 2	*•* multiple-choice (1) *•* single-choice (1) *•* 6-point semantic distance scale (1) *optional:* *•* written responses (qualitative data) (2)	“What further training requirements do you see for yourself in terms of competencies in the use of digital technologies for your teaching and training activities? (Multiple answers possible) Need for further training in: (*basic digital competencies—specific content of certain technologies in nursing practice—pedagogical aspects of integrating technology into teaching*)”
Personal information •*Demography*	5	*•* single-choice (4) *•* written responses (qualitative data) (1)	“How many hours per week do you perform your teaching and training activities? (Average of the last 5 years) (*free text*)”
General remarks	1 *optional:* 1	*•* written responses (qualitative data) (1) *optional:* *•* written responses (qualitative data) (1)	“Why did you choose the paper/digital format of the questionnaire? (*free text*)”

Potential participants were sent an envelope containing a cover letter and the questionnaire in paper format to their official/institutional mailbox address and were asked to send back completed questionnaires via inhouse mail to the Center of Implementing Nursing Care Innovations at the University Medical Center, Freiburg. To increase the reach of the survey, the questionnaire could alternatively be completed online via a link provided in the cover letter. Participants were free to choose the format. A reminder e-mail was sent to all potential participants six weeks after commencement of the survey. Survey data were collected between July and September 2020.

### Analysis

Quantitative data were analyzed using IBM SPSS Statistics for Windows (version 26). Frequencies and percentages were calculated for all quantitative variables. Means and standard deviations (SD) were calculated for interval scaled variables, and medians and interquartile ranges (IQR) for ordinal scaled variables. Explorative subgroup analyses were performed based on job designation, age, and gender. Group comparisons were calculated using Chi-squared test, Fisher’s exact test, Mann–Whitney U test, or Jonckheere’s trend test in combination with Kendall’s tau correlation coefficient. Statistical tests were selected depending on scale level and the number of groups to be compared. Since no normal distribution was assumed and tested, nonparametric tests were used. Statistical significance was set at *p* ≤ 0.05. Qualitative data from open-ended questions were analyzed by means of thematic qualitative text analysis [37] using MAXQDA 20.

Missing data were analyzed descriptively for each item with exception of optional items. The threshold level for noticeable values was set at > 5.0%. Items with missing data or indicated as “not specified” were excluded from the respective analysis.

## Results

A total of *n* = 169 educating nurses participated in the survey (response rate = 52.0%). The majority of the respondents were clinical mentors, female, and completed the questionnaire in paper format. The distribution of the respective age groups appeared nearly split into thirds. Only a small proportion of respondents had more than 15 years of work experience. Clinical mentors were significantly younger than nurse educators. The latter spent significantly more time on teaching and training activities per week than clinical mentors. Detailed sample characteristics are presented in Table 2.

**Table 2:** Sample characteristics and subgroup differences based on job designation

	**Total sample (*n* = 169)** n (%)	**Clinical mentors (*n* = 133)** n (%)	**Nurse Educators (*n* = 28)** n (%)	**Group difference**
**Job designation**				
Clinical mentor	133 (78.7)	-	-	-
Nurse educator	28 (16.6)			
Missing/not specified	8 (4.7)			
**Gender**				
Female	109 (64.5)	83 (62.4)	20 (71.4)	*p* = 0.215^*^
Male	52 (30.8)	46 (34.6)	6 (21.4)	
Others	0 (0)	0 (0)	0 (0)	
Missing/not specified	8 (4.8)	4 (3.0)	2 (7.1)	
**Age** (in years)				
18–35	50 (29.6)	44 (33.1)	4 (14.3)	*p* = 0.001^**,***^
36–49	66 (39.1)	55 (41.4)	7 (25.0)	
≥ 50	47 (27.8)	32 (24.1)	15 (53.6)	
Missing/not specified	6 (3.6)	2 (1.5)	2 (7.1)	
**Work experience in years**				
0–5	70 (41.4)	59 (45.0)	9 (32.1)	*p* = 0.059^**^
6–10	33 (19.5)	28 (21.4)	4 (14.3)	
11–15	31 (18.3)	26 (19.5)	5 (17.9)	
> 15	28 (16.6)	18 (13.5)	9 (32.1)	
Missing/not specified	7 (4.1)	2 (1.5)	1 (3.6)	
**Time spent on teaching and training activities per week in hours** (average of the last 5 years)	*mean (SD; range)* 13.1 (9.93; 1.0–40.0)	*mean (SD; range)* 9.9 (6.8; 1.0–38.5)	*mean (SD; range)* 24.8 (11.5; 3.0–40.0)	*p* = < 0.001^**,***^
**Questionnaire format type**				
Paper	145 (85.8)	117 (88.0)	21 (75.0)	*p* = 0.075^*^
Digital	24 (14.2)	16 (12.0)	7 (25.0)	

^*^Chi-squared test; ^**^Mann-Whitney-U test; ^***^statistically significant; SD = Standard deviation

### Missing values

Three items from three different domains showed rates of missing values > 5.0% (5.3%, 17.2%, 21.9%). Of respondents, 11.2% (*n* = 19) completed all and 74.0% (*n* = 125) > 95% of the items of the questionnaire. Little’s MCAR test did not provide any indication that the missing values were not missing completely at random (*p* = 0.873).

### Digital competence in general

Table 3 shows the results of self-assessments on the statements about general digital competence which can be summarized as follows: 86.3% (*n* = 145) of respondents thought, they knew most of the functions of the electronic devices in their possession; 89.3% (*n* = 151) indicated that it would be easy for them to learn how to operate an electronic device; and 75.3% (*n* = 125) stated that they would be knowledgeable about electronic devices. Nearly 40% (*n* = 67) said that they would have comprehension problems reading a magazine on electronics and computers.

**Table 3:** Self-ratings of statements of the complete study sample and relevant subgroup comparisons in terms of general digital competence with a focus on knowledge

**Statement**	**Response options** *Rating from (1) “does not apply at all” to (4) “fully applies”*	**n (%)**	**Median (IQR)**	**Subgroup comparison (*p*-values)**		
				Job designation^**^ [n]	Gender^**,^^**^ (mean ranks) [n]	Age^***^ (correlation coefficient^****^) [n]
1. I know most of the functions of the electronic devices I own. (*n* = 168)	does not apply at all	0 (0)	3.0 (1.0)	n.s	0.004^*****^ (f: 73.93 < m: 91.54) [160]	0.002^*****^ (-0.225) [162]
	rather does not apply	23 (13.7)				
	rather applies	77 (45.8)				
	fully applies	68 (40.5)				
2. I have or would have comprehension problems reading electronics and computer magazines. (*n* = 168)	does not apply at all	46 (27.4)	2.0 (2.0)	n.s	0.045^*****^ (f: 85.37 > m: 70.38) [160]	0.003^*****^ (0.204) [162]
	rather does not apply	55 (32.7)				
	rather applies	50 (29.8)				
	fully applies	17 (10.1)				
3. It is easy for me to learn how to operate an electronic device. (*n* = 169)	does not apply at all	3 (1.8)	3.0 (1.0)	n.s	0.001^*****^ (f: 76.29 < m: 97.16) [161]	< 0.001^*****^ (-0.283) [163]
	rather does not apply	15 (8.9)				
	rather applies	91 (53.8)				
	fully applies	60 (35.5)				
4. I am knowledgeable about electronic devices. (*n* = 166)	does not apply at all	3 (1.8)	3.0 (1.3)	n.s	0.001^*****^ (f: 71.87 < m: 95.51) [158]	0.018^*****^ (-0.168) [160]
	rather does not apply	38 (22.9)				
	rather applies	97 (58.4)				
	fully applies	28 (16.9)				

^*^comparison between male and female; ^**^Mann-Whitney-U test; ^***^Jonckheere’s trend test; ^****^Kendall’s tau correlation coefficient; ^*****^statistically significant; *IQR* Interquartile range, *y* Years, *f* Female, *m* Male, *n.s.* Not statistically significant

With regard to specific activities in the context of digital technologies, educating nurses could indicate the degree of their competence on a 6-point semantic distance scale from (1) “very bad” to (6) “very good” (Table 4). Means ranged from 4.3 to 5.5. The respondents rated the creation of digital presentations and the connection and use of external devices the lowest. For each specific activity, at least 50% of the respondents considered themselves to be at a high competence level. Additional specific activities mentioned in written responses were “operating ‘office’ software applications”, “conducting video conferences”, and “designing and conducting eLearning”.

**Table 4:** Self-ratings of statements of the complete study sample and relevant subgroup comparisons in terms of specific activities in the context of digital technology (sorted in descending order of mean rating)

**Item (n)** *Items could be rated from (1) “very bad” to (6) “very good”*	**Mean**	**SD**	**Subgroup comparison (*p*-values)**		
			Job designation^**^ (mean ranks) [n]	Gender^*,**^ (mean ranks) [n]	Age^***^ (correlation coefficient^****^) [n]
Print documents (*n* = 169)	5.5	0.674	0.026^*****^ (CM: 77.76 < NE: 96.41) [161]	n.s	n.s
Edit e-mails (*n* = 168)	5.4	0.730	0.002^*****^ (CM: 75.77 < NE: 102.28) [160]	n.s	n.s
Manage data (e.g. create folders, move/copy data) (*n* = 168)	5.1	1.184	0.011^*****^ (CM: 76.54 < NE: 99.15) [160]	n.s	0.001^*****^ (-0.179) [163]
Take digital photos (*n* = 167)	5.0	0.963	n.s	n.s	< 0.001^*****^ (-0.244) [161]
Downloading files from the Internet (*n* = 168)	4.9	1.069	n.s	n.s	n.s
Use word processing (e.g. Word) (*n* = 169)	4.8	1.008	< 0.001^*****^ (CM: 75.10 < NE: 109.04) [161]	n.s	n.s
Comply with data protection rules in the digital world (*n* = 168)	4.6	1.061	n.s	n.s	n.s
Use subject-specific portals on the Internet (*n* = 163)	4.5	1.135	n.s	n.s	n.s
Create a digital presentation (*n* = 169)	4.3	1.435	< 0.001^*****^ (CM: 74.57 < NE: 111.54) [161]	n.s	0.007^*****^ (-0.180) [163]
Connect and use external devices to a PC (e.g. beamer) (*n* = 168)	4.3	1.411	0.001^*****^ (CM: 74.91 < NE: 106.86) [160]	< 0.001^*****^ (f: 71.69 < m: 98.80) [160]	n.s

^*^comparison between male and female; ^**^Mann-Whitney-U test; ^***^Jonckheere’s trend test; ^****^Kendall’s tau correlation coefficient; ^*****^statistically significant; *CM* Clinical mentor, *NE* Nurse educator, *n.s.* Not statistically significant

### Digital competence related to teaching and training activities

One-third (35.1%, *n* = 59) referred to themselves as beginners in terms of their digital competences at a pedagogical-didactic level. Almost two-thirds (61.9%, *n* = 104) considered themselves to be advanced and 3.0% (*n* = 5) as experts. More than half of the respondents (56.3%, *n* = 90) felt that there was an external expectation to incorporate new digital technologies into their teaching and training activities. Supervisors, colleagues, and students were stated as sources of such expectations.

**Fig. 1:**
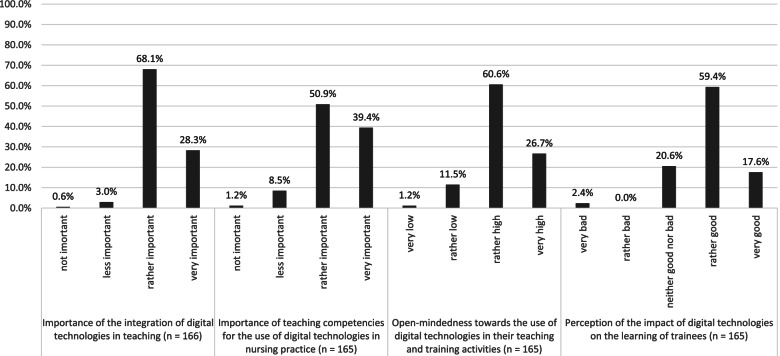
Attitudes of educating nurses towards digital technologies on a pedagogical-didactic level

Figure 1 shows respondents’ attitudes towards digital technology on a pedagogical-didactic level. The vast majority of respondents perceived the integration of digital technologies as a didactic tool (96.4%, *n* = 160) as well as the teaching of competencies for the use of digital technologies in nursing practice (90.3%, *n* = 149) as rather or very important. Predominantly, respondents (87.3%, *n* = 144) considered themselves being open-minded towards the use of digital technologies in their teaching and training activities. Three-quarters of respondents (77.0%, *n* = 127) rated the impact of digital technologies on trainees’ learning as good or very good. However, 20.6% (*n* = 34) were indifferent.

Regarding the digital technology equipment in their workplaces, educating nurses were asked whether they knew various digital technologies, and what the availability and frequency of use in their workplace was (Table 5). The most common unknown technologies (> 10%) were Virtual Reality (VR) glasses, digital blackboards, messenger services, computer labs, and simulation manikins. All other technologies were known to > 90% of respondents. Digital blackboards and tablets were only available to less than 20% of respondents. VR glasses did not exist in any of respondent’s workplace. Based on the median frequency of use, proposed digital technologies were divided into four groups: Those that were used (1) never, (2) seldom, (3) often, and (4) very often.

**Table 5:** Knowledge, availability, and frequency of use of digital technologies in the workplaces of educating nurses (sorted by the Medians of frequency of use; crosslines indicate groups defined by frequency of use)

**Digital technology** (number of respondents)	**Knowledge** n (%)		**available at workplace** n (%^*^)	frequency of use^**^		never used^***^
	unknown	known		n	Median (IQR)	n (%)
Printer (*n* = 168)	1 (0.6)	167 (99.4)	167 (100)	164	4.0 (1.0)	1 (0.6)
E-mail account (*n* = 167)	2 (1.2)	165 (98.8)	164 (99.4)	160	4.0 (1.0)	1 (0.6)
Computer (*n* = 169)	1 (0.6)	168 (99.4)	165 (98.2)	157	4.0 (1.0)	1 (0.6)
Intranet (*n* = 169)	2 (1.2)	167 (98.8)	165 (98.8)	163	3.0 (1.0)	0 (0)
Internet access (*n* = 168)	3 (1.8)	165 (98.2)	161 (97.6)	154	3.0 (1.0)	0 (0)
WLAN (*n* = 167)	2 (1.2)	165 (98.8)	158 (95.8)	153	3.0 (1.0)	5 (3.3)
Subject specific database (*n* = 166)	8 (4.8)	158 (95.2)	137 (86.7)	135	3.0 (1.0)	2 (1.5)
Messenger service (*n* = 163)	27 (16.6)	136 (83.4)	51 (37.5)	49	3.0 (1.0)	4 (8.2)
Digital learning platform (*n* = 161)	15 (9.3)	146 (90.7)	100 (68.5)	97	2.0 (1.0)	3 (3.1)
Projector (*n* = 169)	3 (1.8)	166 (98.2)	90 (54.2)	86	2.0 (2.0)	11 (12.8)
Digital camera (*n* = 168)	5 (3.0)	163 (97.0)	76 (46.6)	76	2.0 (1.0)	9 (11.8)
Simulation manikin (*n* = 166)	17 (10.2)	149 (89.8)	75 (50.3)	75	2.0 (1.0)	16 (21.3)
Smartphone (*n* = 167)	2 (1.2)	165 (98.8)	75 (45.5)	72	2.0 (2.0)	4 (5.6)
Computer lab (*n* = 165)	19 (11.5)	146 (88.5)	51 (34.9)	45	2.0 (1.0)	5 (5.6)
Video conference (*n* = 167)	13 (7.8)	154 (92.2)	48 (31.2)	44	2.0 (0.75)	11 (25.0)
Digital blackboard (*n* = 168)	62 (36.7)	106 (62.7)	29 (27.4)	29	2.0 (0.5)	5 (17.2)
Tablet (*n* = 169)	1 (0.6)	168 (99.4)	22 (13.1)	20	2.0 (1.0)	4 (20.0)
VR glasses (*n* = 166)	69 (41.6)	97 (58.4)	0 (0.0)	0	-//-	-//-

^*^percentage of those respondents that know the digital technology; ^**^Items could be rated from (1) “never”, (2)”seldom”, (3) “often”, (4) “very often”, ^***^corresponds to the rating category (1) "never”; *IQR* Interquartile range

### Requirements and needs

**Fig. 2:**
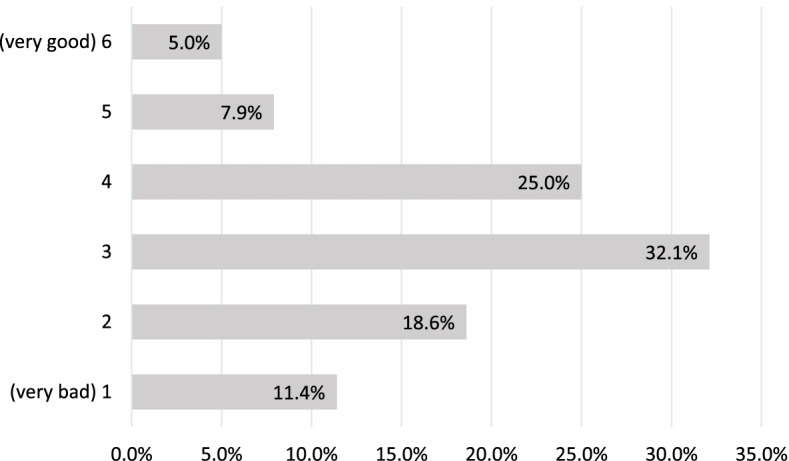
Self-assessment of educating nurses on how prepared they feel to integrate new digital technologies into their teaching and training activities (*n* = 140)

On a 6-point scale, respondents (*n* = 140) were able to specify how prepared they felt to integrate digital technologies into their teaching and training activities. Most responses aggregated in the middle range of the scale (mean = 3.1, SD = 1.28) with a trend toward its lower half (Fig. 2). Content analysis of the respondents’ statements regarding their needs in relation to their teaching and training activities and digital technologies revealed five thematic areas: (1) Training, instruction, and further education, (2) hardware and software equipment, (3) venues, (4) time resources, and (5) contact person.

In relation to further training, respondents indicated their needs mainly on specific content on technologies in nursing practice (69.6%, *n* = 117) and on pedagogical aspects of technology integration in teaching and training activities (71.4%, *n* = 120). Thirty percent (*n* = 51) reported a need for further training on basic digital competencies. Three respondents (1.8%) indicated no need for further training at all. When asked for specific training needs, respondents indicated two overarching topics: (a) Refreshing and consolidating existing competencies and (b) adapting to different levels of competence. Almost all of the respondents (96.3%, *n* = 158) stated that they would attend further training events on the suggested topics.

### Influence of job designation, gender, and age on digital competence—subgroup analysis

In the following paragraphs, relevant statistically significant results of the analysis of three subgroups (job designation, gender, and age) are presented.

Concerning *job designation* there was no difference in their self-ratings on digital competence in general with a focus on knowledge between nurse educators and clinical mentors (Table 3). However, in six out of ten activity descriptions nurse educators showed higher self-ratings (Table 4). The proportion of those individuals who felt an external expectation to incorporate new digital technologies into their teaching and training activities was nearly twice as high among nurse educators (89.3%, *n* = 25) as among clinical mentors (47.6%, *n* = 60) (χ^2^(1) = 16.1; *p* < 0.001). Nurse educators found it more important to teach competencies for the use of digital technologies in nursing practice compared to clinical mentors (mean ranks 93.93 vs. 75.90; *p* = 0.036) and more often knew computer labs, digital blackboards, and simulation manikins (Table 6). These technologies, together with digital learning platforms, projectors, smartphones, and video conference equipment, were more frequently available at nurse educators’ workplaces and therefore used more frequently by nurse educators. Exceptions were simulation manikins and digital blackboards; both technologies were used more frequently by clinical mentors. However, data on digital blackboard usage is based on only two clinical mentors and the result might therefore be biased (Table 6). Clinical mentors more often stated the need for further training on specific content on (new) technologies in nursing practice (χ^2^(1) = 26.9; *p* < 0.001).

**Table 6:** Results of the subgroup analysis concerning knowledge, presence, and frequency of use of digital technologies in the workplaces of educating nurses

**Digital technology**		**Subgroup comparisons (*p*-values)**					
	**Job designation**			**Gender**^a^		**Age**	
	Knowledge^b^ [n]	available at workplace^b^	frequency of use^c^ (mean ranks) [n]	Knowledge^b^ [n]	frequency of use^c^ (mean ranks) [n]	knowledge^d^ (correlation coefficient^e^) [n]	frequency of use^d^ (correlation coefficient^e^) [n]
Printer	n.s	n.s	0.022^f^ (CM: 75.12 < NE: 94.67) [156]	n.s	n.s	n.s	n.s
E-mail account	n.s	n.s	0.001^f^ (CM: 72.52 < NE: 98.88) [153]	n.s	n.s	n.s	n.s
Computer	n.s	n.s	0.014^f^ (CM: 72.20 < NE: 91.23) [150]	n.s	0.003^f^ (f: 81.76 > m: 63.71) [150]	n.s	n.s
Intranet	n.s	n.s	n.s	n.s	n.s	n.s	n.s
Internet access	n.s	n.s	0.006^f^ (CM: 70.37 < NE: 93.02) [148]	n.s	n.s	n.s	n.s
WLAN	n.s	n.s	n.s	n.s	n.s	n.s	n.s
Subject specific database	n.s	n.s	n.s	n.s	n.s	n.s	n.s
Messenger service	n.s	n.s	n.s	n.s	n.s	0.021^f^ (-0.175) [157]	n.s
Digital learning platform	n.s	< 0.002^f^ (CM < NE) [153]	0.001^f^ (CM: 42.60 < NE: 63.54) [94]	n.s	n.s	n.s	n.s
Projector	n.s	< 0.001^f^ (CM < NE) [161]	< 0.001^f^ (CM: 29.73 < NE: 61.02) [81]	n.s	n.s	n.s	0.001^f^ (0.315) [81]
Digital camera	n.s	n.s	n.s	n.s	n.s	n.s	n.s
Simulation manikin	0.044^f^ (CM < NE) [158]	< 0.001^f^ (CM < NE) [158]	0.013^f^ (CM: 39.29 > NE: 28.23) [70]	n.s	0.004^f^ (f: 31.35 < m: 44.55) [70]	n.s	n.s
Smartphone	n.s	0.032^f^ (CM < NE) [131]	n.s	n.s	n.s	n.s	n.s
Computer lab	0.026^f^ (CM < NE) [157]	< 0.001^f^ (CM < NE) [157]	n.s	n.s	n.s	n.s	n.s
Video conference	n.s	< 0.001^f^ (CM < NE) [159]	n.s	n.s	n.s	n.s	n.s
Digital blackboard	< 0.001^f^ (CM < NE) [160]	< 0.001^f^ (CM < NE) [160]	0.023^f^ CM: 24.25 > NE: 12.60) [26]	n.s	0.003^f^ (f: 12.80 < m: 20.88) [26]	n.s	n.s
Tablet	n.s	n.s	n.s	n.s	n.s	n.s	n.s
VR glasses	n.s	-//-	-//-	0.001^f^ (f < m) [158]	-//-	0.002^f^ (-0.226) [160]	-//-

^a^comparison between male and female; ^b^Fisher’s exact test or Chi squared test, respectively; ^c^Mann-Whitney-U test; ^d^Jonckheere’s trend test; ^e^Kendall’s tau correlation coefficient; fstatistically significant; *CM* Clinical mentor, *NE* Nurse educator, *f* Female, *m* Male, *y* Years, *n.s.* Not statistically significant

Female *gender* was associated with a lower self-perceived general digital competence with a focus on knowledge (Table 3). In terms of skill, the self-ratings only differed in connecting and using external devices to a PC with higher ratings in males (Table 4). Males knew VR glasses more often, and more frequently used digital blackboards and simulation manikins. Computers were more often used by females (Table 6). Female compared to male respondents (mean ranks 62.96 vs. 82.21; *p* = 0.007) felt less prepared to integrate new digital technologies into their teaching and training activities and showed a higher commitment to attend further training events (χ^2^(1) = 7.9; *p* = 0.012).

Younger *age* was associated with higher self-perceived general digital competence in all knowledge focused and three skill-focused statements (Tables 3 and 4). Messenger services and VR glasses were more often known by younger respondents, whereas projectors were used more frequently by older respondents (Table 6). Older age was associated with perceiving external expectations to incorporate new digital technologies into teaching and training activities more frequently (*p* = 0.004; τ = -0.217) but feeling less prepared to do so (*p* = 0.002; τ = -0.220) with a simultaneous lower degree of open-mindedness towards the use of digital technologies in teaching and training activities (*p* < 0.001; τ = -0.290). Moreover, older age was associated with a greater need to take part in trainings of basic digital skills (*p* = 0.005; τ = -0.210).

## Discussion

A survey in two hospitals and affiliated nursing schools in Germany was conducted to explore digital competence, needs and requirements of educating nurses concerning the digital aspects of their pedagogy and teaching activities. In this survey, educating nurses rated themselves rather high in terms of their general digital competence and predominantly as being advanced in terms of digital competence related to teaching activities. Overall, respondents indicated positive attitudes towards digital technology on a pedagogical-didactic level, and, mostly, a basic configuration of digital teaching technology seemed to exist at the respective workplaces. Nevertheless, many respondents indicated a need for further training in and about digital technologies in the context of teaching.

Similar to the findings of Ryhtä et al. [17], the self-assessments of digital competence in terms of both knowledge and skill were at a very high level. This provides a crucial basis for further development of digital competence and its application in a professional context [6, 29]. A lower self-assessed level of competence was observed in reading computer magazines, which could be linked to the higher degree of abstraction of this statement.

Only two activity descriptions showed low competence level rates slightly more than 10%, i.e. to connect and use external devices to a PC and to create a digital presentation. In the view of the researchers of this study, the ability to connect digital devices is a basic skill for using devices and technologies effectively. In the questionnaire, this item differs from the others in that it is a skill that has both an analogous (practical-haptic) and a digital component. This could possibly have led to the somewhat poorer rating. Interestingly, it was the only activity description with a statistical difference between genders. The low competence level rate in the creation of a digital presentation, is particularly surprising given that this is a common and widespread teaching medium [38]. However, the sample consisted mainly of clinical mentors, who were less likely to use this technology, and showed a statistical significant lower competence self-rating compared to nurse educators. Overall, it was noticeable that the knowledge-focused digital competence assessment was characterized by gender and age differences, while the skill-focused digital competence assessment was in turn distinguished by job designations.

The high level of self-assessment of digital competence was also reflected in the area of professional teaching and training. Most respondents rated themselves as “advanced” and were familiar with almost all of the technologies mentioned. This indicates that they had already dealt with and applied digital technology in a professional context, which parallels the results of Nguyen et al. [32] where the majority of the teaching nurses rated themselves as "advanced beginner” or “competent" with regard to new technologies for education and practice. More frequently unknown technologies tended to be either generally new and not yet widely disseminated ones, e.g. VR glasses [39], or setting-specific, such as digital blackboards and computer labs in nursing schools. Surprisingly, despite their global popularity, messenger services were among these frequently unknown technologies, which could probably be explained by not using a brand name in the questionnaire.

One third of the proposed technologies were present in workplaces of nearly all respondents, and thus were used frequently. These technologies could be described as "classic" digital technologies or standard equipment. Nearly half of the remaining technologies were again dependent on the professional setting and more common in nursing schools and more often used by nurse educators. A possible explanation could be that many of the technologies available for selection in the questionnaire are more suitable for theoretical training in teaching at nursing schools, e.g. projector. They seem to be unsuitable for practical training during placements, which often happens in dyads. The diffusion of technologies in the clinical setting in Germany is in full swing, but these technologies focus primarily on patient care [40] and less, as in this study, on the education of nurses. Finally, in the theoretical training setting, nursing students are more likely to have technical devices, such as smartphones or laptops, with them [41]. This may lead to a higher likelihood of the use of digital technologies in teaching on the part of nurse educators. On the other hand, this may also increase the external expectation on teachers to integrate technology [20], which was also perceived by the respondents—particularly by nurse educators and older respondents—in the sample of the present study.

Of note were the results on tablets. While the vast majority of respondents knew this technology, only a minority had access to it at the workplace. Given the evidence supporting the benefits of tablets for health care professional education [42] and patient care [43], there may be a potential for increased use of this mobile technology in workplaces [44].

The least used technology, despite its availability, were video conferencing systems. Although video conferencing can be considered a common technology, it has only been used increasingly since the Covid-19 pandemic [45]. Whilst these are activities that can be performed remotely, e.g., distance learning, they do not include direct care activities with the exception of telehealth, which is not yet widespread in Germany [46].

Use and integration of digital technologies in teaching and training is also determined by the attitude of teachers [47]. The respondents in the present study showed a positive attitude toward digital technologies in the teaching context. Despite this positive attitude and good self-assessments regarding digital competencies, many respondents did not feel adequately prepared to integrate new digital technologies into their teaching. This is in contrast to the study by Talcott et al. [48] which stated that 76% of nurse educators surveyed in the U.S. felt prepared using learning technologies in teaching. However, cultural differences cannot be disregarded here. Female and older respondents in particular felt inadequately prepared.

Continuing education and training are ways of acquiring and expanding digital competencies. Almost all respondents in this survey expressed a need for further training concerning their digital competencies and were motivated to take up such offers. However, attention should also be paid to the differences in the subgroups analyzed. Age and gender are two central factors associated with digital competence [49]. In other studies on digital competencies in healthcare contexts, it is primarily older age that is often described as a factor with an inverse relationship to digital competence [8, 34]. In the present study, differences between younger and older or female and male respondents were more likely to be observed in general digital competencies, the perceived preparedness for teaching using digital technology, or different needs for further training. Nurse educators and clinical mentors were more likely to differ in terms of equipment and frequency of use of digital teaching technologies. However, a clear pattern cannot be discerned and was not the aim of this exploratory analysis. More in-depth analyses are necessary to guide decision making whether it seems reasonable to design subgroup-specific interventions. Finally, the very high motivation suggests that adequate training and support offers tailored to the needs of educating nurses would be embraced [20].

### Strengths and limitations

This survey achieved a high response rate and a low rate of missing values. Combined with the sample size, this provides a detailed evidence base for deriving initial ideas for possible interventions. Nonetheless, the sample consisted of more than four times as many clinical mentors as educators. Although this may be close to the real-world ratio, the results of this survey are more influenced by the assessments of clinical mentors. Subgroup analysis was in some cases characterized by an imbalanced and a small number of respondents requiring the results to be interpreted with caution. Moreover, the results of the present study are based only on a convenience sample in a regional setting and cannot be generalized for all educating nurses in Germany. In addition, self-reporting questionnaires may lead to biased, over- or underestimated responses influenced by social desirability. Finally, data collection instrument used in the present study appears to be only face valid, which limits drawing conclusions.

## Conclusions

This survey demonstrated that educating nurses feel digitally competent in general and advanced in terms of teaching and training activities. Positive attitudes towards digital technology on a pedagogical-didactic level and the existence of a basic configuration of digital teaching technology in the sample setting suggest a good starting point for the use and further development of digital teaching in nursing education. The need for further training in and about digital technologies in the context of teaching indicated by the respondents and the results of the subgroup analyses suggesting a gender and age gap demand for action and could be addressed through targeted interventions, e.g. continuing education. The results of this study can be used in the context of personnel development or training of educating nurses as a basis or guidance to adapt already existing training programs or curricula. In addition, the results presented may contribute to the development of a target group-specific instrument for assessing digital competencies of educating nurses. However, further research is needed to deepen and further explore these findings for appropriate intervention development. In the sense of an explanatory sequential design [50] this could be accomplished by qualitative interviews with representatives of the target group.

## Data Availability

The datasets generated and/or analyzed during the current study are not publicly available but are available from the corresponding author on reasonable request.

## Abbreviations

ICT: Information and communication technology
PC: Personal computer
SD: Standard deviation,
IQR: Interquartile range
STROBE: Strengthening the reporting of observational studies in epidemiology
